# From Cells to Virus Particles: Quantitative Methods to Monitor RNA Packaging

**DOI:** 10.3390/v8080239

**Published:** 2016-08-22

**Authors:** Mireia Ferrer, Simon Henriet, Célia Chamontin, Sébastien Lainé, Marylène Mougel

**Affiliations:** 1 CPBS, CNRS, Université de Montpellier, 1919 Route de Mende, Montpellier 34293, France; mireia.dream@gmail.com (M.F.); celia.chamontin@cpbs.cnrs.fr (C.C.); sebastien.laine@cpbs.cnrs.fr (S.L.); 2 Sars International Centre for Marine Molecular Biology, University of Bergen, Bergen 5018, Norway; Simon.Henriet@uib.no

**Keywords:** RNA, packaging, retrovirus, HIV-1, assembly, Gag, fluorescence microscopy, RNA imaging, RT-qPCR, Northern blot

## Abstract

In cells, positive strand RNA viruses, such as *Retroviridae*, must selectively recognize their full-length RNA genome among abundant cellular RNAs to assemble and release particles. How viruses coordinate the intracellular trafficking of both RNA and protein components to the assembly sites of infectious particles at the cell surface remains a long-standing question. The mechanisms ensuring packaging of genomic RNA are essential for viral infectivity. Since RNA packaging impacts on several essential functions of retroviral replication such as RNA dimerization, translation and recombination events, there are many studies that require the determination of RNA packaging efficiency and/or RNA packaging ability. Studies of RNA encapsidation rely upon techniques for the identification and quantification of RNA species packaged by the virus. This review focuses on the different approaches available to monitor RNA packaging: Northern blot analysis, ribonuclease protection assay and quantitative reverse transcriptase-coupled polymerase chain reaction as well as the most recent RNA imaging and sequencing technologies. Advantages, disadvantages and limitations of these approaches will be discussed in order to help the investigator to choose the most appropriate technique. Although the review was written with the prototypic simple murine leukemia virus (MLV) and complex human immunodeficiency virus type 1 (HIV-1) in mind, the techniques were described in order to benefit to a larger community.

## 1. Introduction

Most retroviruses share the same characteristics: a spherical capsid about 110–120 nm in diameter enclosing a positive single-strand RNA genome. During infection, the genomic RNA (gRNA) is retro-transcribed into DNA, which is integrated into the host chromosomes. Transcription of this viral DNA gives rise to a primary, full-length transcript that acts as gRNA for packaging into progeny viruses. In addition, the gRNA serves as a messenger RNA (mRNA) template for the synthesis of the structural Gag and enzymatic Pol polyproteins. A common feature of most retroviruses is the selective packaging among the bulk of viral spliced and cellular RNAs of two identical copies of gRNA per viral particle. The packaging of retroviral RNA is a highly regulated process that requires structural motifs located in the 5’ untranslated region of gRNA, notably the Psi region. Psi folds into stem-loops that constitute the major packaging determinants recognized by Gag to be packaged into virions (for review, see [1,2]). These determinants contribute also to the dimerization of the gRNA, a prerequisite for gRNA packaging [3,4]. However, it is now well-accepted that virions can specifically incorporate several other RNA species in addition to the gRNA. Indeed, several small RNA polymerase III (Pol-III) generated RNAs such as transfer RNA (tRNA), 5S ribosomal RNA (rRNA), U6 small nuclear RNA (snRNA), mY RNAs, 7SL RNA and 7SL RNA-derived short interspersed nuclear elements (SINE) RNAs (B1 RNA or Alu RNA) are enriched into virions. For unknown reasons, the U6 snRNA is enriched, while U1 and U2 snRNAs are found only in trace amounts. 7SL RNA and U6 snRNA are not randomly packaged but are recruited in human immunodeficiency virus type 1 (HIV-1) and murine leukemia virus (MLV) through a selective mechanism [5,6,7,8,9]. Interestingly, complementary DNA (cDNA) copies of these two cellular RNA species have been found into the host cells [10,11], suggesting that they are part of infectious particles. There are assumptions for functional implications of the cellular RNAs in the retrovirus lifecycle [12].

Although the *cis*- and *trans*-acting elements of RNA packaging are well-documented (for review, see [12,13]), many aspects of the RNA-RNA interactions and RNA selection are still unclear because these questions cannot be answered using conventional biochemical or microscopy approaches. Interestingly, RNA imaging experiments on the scale of single RNA molecules have been recently developed and provide powerful tools to elucidate the mechanisms of RNA packaging. For instance, RNA recruitment for packaging has been visualized in cells (for review, see [1]) and multicolor total internal reflection fluorescence microscopy (TIRFM) techniques enabled the detection of gRNA dimers at the plasma membrane [14,15]. Further insights have been gained using two advanced single-molecule fluorescence microscopy approaches, super-resolution and the number and brightness (N&B) method. Indeed, the combination of these two techniques provided a three-dimensional vision of cells revealing, for the first time, the presence of HIV-1 gRNA dimers in the cytosol [15]. These new data support the notion that viral RNA competent for packaging is selected early in the cytoplasm. In the case of simple retroviruses, the RNA could be selected even earlier. Indeed, there is some indirect evidence suggesting that MLV, could initiate gRNA dimerization in the nucleus [16,17,18].

In order to understand the fundamental mechanisms of retroviral replication, it is essential to accurately characterize the identity and amount of RNA packaged into virions. This includes not only the gRNA, the key molecule carrying all the viral genetic information, but also RNA from the host cell, which may contribute to the viral lifecycle [12]. The present review will not address the delicate issue of virus purification, whose main challenge is to efficiently sort out retroviral particles from cellular exosomes that might also contain cellular RNAs [19]. Classical approaches for studying viral packaging rely on RNA extraction and purification from biological samples and thus necessitate appropriate methods for the best recovery of target RNA. Depending on the analysis being performed, one may choose either unbiased approaches aimed at recovering the whole population of RNA, or selective purification of classes of interest such as poly-adenylated transcripts. When addressing how efficiently RNA is selected into virions, two notions must be defined: the calculation of packaging efficiency and the RNA packaging ability. The packaging efficiency is the absolute amount of RNA per virus or virus-like particle (VLP) and is therefore measured with quantitative methods that monitor both the intravirion RNA amount and level of released viral particles. The most precise approach for determining viral production is based on the combination of commercial enzyme-linked immunosorbent assay (ELISA) assays for Gag (capsid) and measurement of reverse transcriptase activity. The RNA packaging ability corresponds to the ratio of gRNA amount in virions or VLP to gRNA in cells (gRNA virus/gRNA cell). This ratio relies only on RNA (and not virion protein) quantification since the gRNA molecule is always associated to viruses or VLP and not secreted alone in the extracellular media. We will describe the different approaches available to quantify RNA in cells and virions. They are based on conventional biochemical analyses and fluorescence imaging systems. Some techniques can also provide useful additional information, for instance about the structure and the localization of the molecule being detected. We will describe the technical concepts on which these different methods are based, and how they permit the study of RNA packaging. The advantages and limitations will be discussed in terms of performance, accuracy and practical use in the laboratory.

## 2. In Vitro Gel-Based Approaches: Northern Blot and Ribonuclease Protection Assay

**Figure 1:**
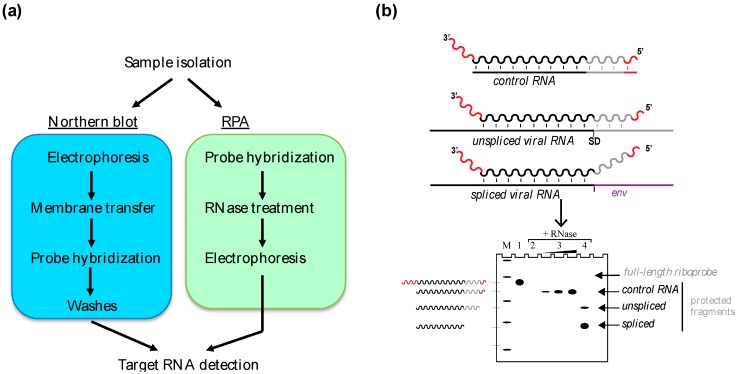
Gel-based detection of RNA. (**a**) Comparison of the experimental workflow in Northern blot (left) and ribonuclease protection assay (RPA) (right). Several steps are common to both techniques, including probe labeling, hybridization and electrophoresis. (**b**) Schematic representation of probe annealing during RPA. A synthetic probe is composed of sequences present (black and gray) or absent (red) from the target RNA. The drawing represents probe annealing to a control RNA produced in vitro and to the products of splicing in a simple retrovirus. SD, splice donor site; *env*, envelope gene. Annealing (vertical lines) protects from RNase treatment, generating double-stranded fragments, which are visualized after gel electrophoresis (Lanes: M, size marker; 1, untreated probe; 2, probe only; 3, probe with increasing concentration of control RNA; 4, probe with sample containing retroviral RNA).

The Northern blot and the ribonuclease protection assay (RPA) were among the first methods developed for measuring gene expression in cells and tissues. These techniques use complementary labeled probes for detecting RNA in a purified sample. For detecting long transcripts such as viral mRNA and gRNA, the probe is most often an internally labeled riboprobe, which has been obtained by using radioactive in vitro transcription. The signal is recorded only after several steps which obligatorily include gel electrophoresis (Figure 1a, for a detailed overview of the procedures, see [20]). There is no signal amplification, and RNA integrity is therefore critical when studying low-abundant targets with these techniques. Despite their shortcomings, the Northern blot and the RPA have unique features which make them interesting tools for studying viral RNA expression and packaging.

### 2.1. The Northern Blot

The Northern blot is the RNA equivalent of the Southern blot procedure, a pioneer technique developed for studying DNA fragments. The sample is first run on an electrophoretic gel, usually under denaturing conditions, and then transferred onto a membrane (Figure 1a). After cross-linking the membrane is exposed to a labeled probe complementary to the target RNA, within containers such as bottles or sealed bags. The signal is revealed after successive washes to remove excess, non-specifically bound probe. Indeed, probes can associate to molecules unrelated to the target, such as ribosomal RNA abundant in some samples, and they can also interact with the membrane itself. For these reasons, the optimization of Northern blot assays often consists in finding a compromise between the stringency of washes and the preservation of signal intensity. Annealing and washing conditions for selecting specific interactions between the probe and its target generally include high temperature, denaturing agents and the presence of competitor RNA. Probe design can have dramatic effects on the quality of the detection. Long probes are more susceptible to create background, but they have a higher specific activity, which may be useful for sensitive detection of rare transcripts.

The main advantage of the Northern blot resides in providing information about the length of the target RNA. The assay has thus been widely used during the initial characterization of diverse RNA species, including mRNA, several viral RNA and small interfering RNA. However, the technique presents some significant disadvantages. First of all, the Northern blot protocol is long and it involves the manipulation of large amounts of buffer, some of which is radioactive. The labor-intensive procedure is poorly suited for large-scale studies, even though membranes can be stripped and reprobed to some extent. The membrane transfer step may have adverse effects on the reproducibility and the precision of detection. Indeed, transfer efficiency varies between different RNAs, and a fraction of the sample can remain trapped in the electrophoretic gel. The membrane itself constitutes a poor support for quantitative assays, and some molecules may become inaccessible upon cross-linking to the membrane. Finally, the sensitivity of the Northern blot is critically affected by RNA degradation, as cleaved targets will spread the signal during electrophoresis. Despite its unique advantage for direct estimation of RNA size, the Northern blot is usually not a competitive alternative for accurate measurement of RNA concentration.

### 2.2. The RPA

During the RPA procedure, the probe is added directly to the hybridization buffer containing the sample. Unlike blot hybridization, annealing in solution permits duplex formation with the totality of target RNA. A mixture of RNases specific for single-stranded RNA (usually RNase A and T1) is then added, whose purpose is the removal of excess probe and unprotected fragments—i.e., not engaged in a duplex. Following RNase treatment, the sample is run on a denaturing polyacrylamide gel electrophoresis (PAGE) and the electrophoretic profile of the probe is recorded [21] (Figure 1a). The signal intensity from the protected fragments being proportional to the amount of complementary sequences in the sample, the RPA enables direct measurement of RNA concentration. In contrast to Northern blot, target size can be inferred only in cases where the RNA of interest is smaller than the probe itself [22].

The performance of RPA critically depends on the quality of probe detection with PAGE analysis. Probe length for RPA is most often comprised between 100 and 700 nt, the upper size being usually constrained by the resolution of protected fragments during PAGE analysis. The integrity of the probe being used is the first important parameter to consider. Therefore, it is often required to gel-purify riboprobes after radioactive labeling in order to get rid of extra bands caused by abortive transcription products. We will review some of the issues usually encountered with RPA, and how to address them in order to improve the sensitivity, the specificity and the scale of the assay.

#### 2.2.1. Sensitivity

The RPA provides a significant improvement to sensitivity compared to the Northern blot, partly because it is much less affected by RNA degradation. Indeed, if the target sequence is cut by an endonuclease during sample preparation, the resulting fragments still have a chance to anneal and protect the probe. In addition, solution hybridization permits the testing of large amount of RNA (up to 100 µg for most protocols), whereas Northern blot is limited by the capacity of electrophoresis wells. Substantial improvements to sensitivity can be further obtained with high-specific activity probes, obtained for instance by increasing labeling efficiency. During optimization of the RPA assay, the sensitivity and the linearity of detection can be tested by annealing the probe to serial dilutions of an artificial complementary RNA. Such standards may subsequently be used to infer the absolute concentration of target RNA in the experimental sample (Figure 1b). They may also inform on target RNA present in excess amount, which is usually solved by diluting the sample.

#### 2.2.2. Specificity and Accuracy

The RPA is a highly reproducible method, and it is much less affected by trace contaminants than polymerase chain reaction (PCR) approaches. However, the interaction of the probe with sequences that do not belong to the target RNA can create undesirable protected fragments. They can be caused for example by partially related RNA present in the sample, or by self-complementarity within the probe. Long probes have a higher risk to form stable secondary structures and repeats, which can be addressed by increasing the stringency of annealing conditions. If the RNase treatment is not efficient, extra bands, corresponding to the undigested probe, may appear and this is usually solved by testing different mixtures and concentrations of enzymes. Finally, the RNase treatment might be able to discriminate between single-nucleotide variants [23], which can complicate the analysis of samples carrying some level of polymorphism. Problems related to specificity can eventually be addressed by checking if two distinct probes directed against the same target provide the same protection intensity.

#### 2.2.3. Multiplexing

The concentration of target RNA can be expressed relative to another RNA whose level remains constant. This can be achieved in RPA by multiplexing the number of probes used in the assay [24]. Several different probes can be used simultaneously in the same tube, but the experiment has to be carefully prepared in order to avoid cross hybridization between probes and for obtaining the best separation of protected fragments on PAGE. RPA experiments with multiple probes have been used for testing the level of expression of viral RNA compared to several cellular RNA [25,26].

### 2.3. Applications of Northern Blot and RPA

By combining Northern blot and RPA, it is possible to get exhaustive information about the size, the exon arrangement and the extremities of RNA molecules. A major advantage of these techniques over others is that only partial knowledge of the target RNA sequence is required to design probes. For instance, a Northern blot using a probe corresponding only to the 5’ leader of retroviral RNA, could in theory reveal several variants corresponding to alternatively spliced RNA. In addition, RPA permits researchers to examine how the RNA sequence is arranged at a precise location, for instance splicing sites (Figure 1b) and RNA ends. In contrast, PCR-based approaches necessitate a minima the knowledge about two distal sequences present on the target molecule and the distance between them. Whereas in RPA, any kind of sequence variants can be successfully detected at the location of the probe, in reverse transcriptase-coupled polymerase chain reaction (RT-PCR) a small variation at only one primer location may be sufficient to prevent detection.

The Northern blot and RPA approaches are especially useful for studying RNA viruses since a single probe can simultaneously detect retroviral gRNA and its differentially spliced variants (Figure 1b). For instance, the northern analysis of total RNA sample of cells infected with MLV permitted the discovery of a new viral alternatively-spliced RNA (called SD’) [27] and to confirm its presence in virions. Northern blot was used originally to obtain a rough estimate of the relative abundance of SD’ RNA compared to gRNA, but its precise quantification necessitated other methods [28].

The RPA proved to be a useful tool for testing the packaging efficiency of gRNA mutants. It has been instrumental for deciphering the role played by *cis*-acting sequences during MLV or HIV-1 assembly [29,30,31]. The multiplexing ability permitted to compare the packaging efficiency of viral RNA mutants relative to controls such as co-packaged cellular RNA (i.e., 7SL and U6) or a reporter [12,32]. However, the sensitivity of RPA lies well under quantitative reverse transcription PCR (RT-qPCR) and viral RNAs have often been studied with both techniques. For example, the packaging ability of HIV-1 trans-activation response element (TAR) mutants has been studied with RT-qPCR [6], semi-quantitative PCR, and RPA [33]. While the combination of both approaches can greatly benefit to the specificity of detection, conflicting results can also be observed. This could be the case when the RPA probe and the PCR primers are directed towards distinct regions of the target RNA, which do not have the same stability.

## 3. Quantitative RT-PCR

The RT-qPCR is the method of choice to quantify RNA expressed at very low levels. It is highly specific and much more sensitive than the methods mentioned above. Its fundamental principle is based on monitoring the accumulation of the target amplicon during successive cycles of PCR. Different kinds of approaches based on fluorescence dyes permit to label the double-stranded DNA (dsDNA) being synthesized. During the exponential phase, the amount of the fluorescent signal generated is proportional to the concentration of the target RNA in the original sample. For each PCR reaction, the threshold cycle (*C*t) is defined as the cycle number at which the fluorescent signal of the reaction crosses the threshold. The threshold corresponds to a fluorescence intensity significantly over the background value, which is determined for each sample during the initial cycles of PCR. Differences in threshold cycle number are used to quantify the relative amount of PCR target contained within each tube, and for each experimental sample the initial concentration of target varies inversely with the *C*t value.

### 3.1. Absolute versus Relative Quantification

In absolute quantification, users can determine the exact copy number of target sequences initially present in the sample. For this purpose, a dilution series of a known template similar to the target is amplified with the same qPCR conditions and the results are used to establish a standard curve. The curve is obtained by plotting the log of each concentration in the dilution series against its corresponding *C*t value.

Plasmid DNA or in vitro transcribed RNA are commonly used to prepare absolute standards. Plasmids are very stable and generate highly reproducible standard curves even after a long storage time, whereas freshly synthesized RNA is sensitive to degradation. To mimic RT-qPCR experimental conditions, plasmid dilutions can be prepared in a solution containing total RNA from cells and RT buffer [28]. However, plasmid-based calibration curves provide standardization based solely on the qPCR step. They neglect the RT step, which is needed for converting the target RNA in cDNA capable of being amplified with qPCR. In order to control the efficiency of the reverse transcription, standard RNA can be generated by in vitro transcription of a linearized plasmid, and then used for establishing standard curves after RT and qPCR steps [5,34]. Finally, analyzing the slope of standard curves informs about the reaction efficiency, which has a critical influence on the accuracy and the reproducibility of the assay.

In relative quantification, users monitor changes in a target RNA expression relative to a reference gene. The reference is often the transcript of a housekeeping gene retrotranscribed and co-amplified in the same tube. One approach is to establish standard curves for both target and control RNA. The curves are used to determine the amount of RNA of interest in each sample, which is then normalized to the internal control RNA. Then, the normalized numbers are compared between samples to obtain a fold change. To calculate relative packaging efficiency of the mouse mammary tumor virus (MMTV) RNA for example, β-actin mRNA was used as an endogenous control, thanks to its consistent presence inside the virus particles [35].

### 3.2. Choice of Fluorescence Approach

A variety of fluorescence-based strategies exists to monitor dsDNA accumulation on qPCR. The two most popular ones are the TaqMan Assay, using a fluorogenic probe specific to the target gene [36]; and the SYBR Green assay [37], which is based on a dsDNA binding dye. The TaqMan assay relies on the hybridization of a specific oligonucleotide probe carrying a fluorophore and a quencher at each extremity. TaqMan probe melting temperature (Tm) should be 10 °C higher than the Tm of the primers used for PCR. Then, the PCR-primers extension displaces the probe which is cleaved by the 5′ to 3′ exonuclease activity of the *Taq* polymerase. Degradation of the probe releases the fluorophore and takes it away from the quencher, allowing fluorescence of the fluorophore. TaqMan performs better than SYBR Green assay in terms of sensitivity and specificity, as the fluorescence is only generated when the probe is released to the target sequence during PCR amplification. However, the approach is less economical for multiple target RNA analysis. In contrast, SYBR Green can be used with any pair of primers for any target. Nonetheless, when used during a PCR reaction, SYBR Green will bind to any dsDNA including the template, primer dimers, specific PCR products and nonspecific products. This is a major disadvantage, which can compromise the specificity of detection. There are a few ways of handling this problem, including analyzing DNA melting curves post-PCR and using approaches to reduce mis-priming.

### 3.3. Priming Strategy

Good primer design is one of the most important parameters in real-time PCR for obtaining high specificity and sensitivity. Primer pairs should have compatible melting temperatures and amplicon lengths should be chosen as short as possible (less than 300 pb). The formation of primer-dimers should be avoided, as it decreases PCR efficiency and obscures analysis. Techniques such as hot start reduce mis-priming by suppressing the activity of PCR enzymes at lower temperatures, where non-specific duplexes can form.

When SYBR Green is used, the specificity of the real-time PCR reaction and the absence of primer dimers can be verified using melting curve analysis. Immediately after the PCR cycles, the temperature is raised by increment until all dsDNA molecules are “melted” in single-stranded DNA (ssDNA). Individual amplicons and primer dimers have their own melting temperature. Therefore, by monitoring the change in fluorescence triggered by the release of SYBR Green upon melting, one can estimate the proportion of specific PCR products in the reaction [38].

### 3.4. Quality of Templates

The RT-qPCR is a very sensitive technique capable to detect few copies of transcripts in a complex mixture. This sensitivity is critically dependent on the efficiency of the amplification and on the quality of the cDNA template. Ultrapure, intact RNA is essential for full-length, high quality cDNA synthesis.

Following DNase treatment on nucleic acid extracts to remove DNA contamination, equal RNA amounts are set for reverse transcription. Two negative controls of RT-PCR reactions must be systematically performed: one to confirm the absence of DNA by running a RT reaction without enzyme, and another to evaluate background amplification level by running a real-time PCR with a sample lacking the target RNA, extracted for example from mock-infected cells. 

Oligo(dT) primers are a favorite choice for cDNA synthesis as they prime all RNAs having poly-A 3′ tail simultaneously. It is convenient for viral and cellular mRNAs, while reverse transcription of non-coding RNA may require specific internal or random primers [8,9]. Specific, internal primers corresponding to a single target may provide enhance sensitivity by restricting the RT activity to a single RNA [39]. However, this approach does not provide the same flexibility as oligo (dT) and random primers, and it considerably reduces the diversity of the cDNA library. Priming with random oligomers offers some advantages, as they can prime throughout the entire length of RNA and are not biased toward specific genes or polyadenylated transcripts [5,32]. This provides a more representative cDNA library, but the lack of selectivity can also introduce problems in terms of specificity.

Stable secondary structure elements present in viral RNA may lead to incomplete cDNA generation. Conditions of RT extension, such as temperature, duration of the incubation and choice of the enzyme, can be optimized to guarantee a successful cDNA synthesis [27].

### 3.5. RT-qPCR to Monitor Retroviral RNA Encapsidation

The RT-qPCR has been useful for characterizing the content of retroviral particles and for precisely measuring RNA packaging ability. Two qPCR strategies, TaqMan [5,32,35,40] and SYBR Green [8,28,34] have been successfully used. The encapsidation of viral full-length, singly-spliced and fully spliced RNA into HIV-1 and MLV particles has been determined by RT-qPCR [5,8,28]. With specific primers, it was also possible to monitor the abilities of some cellular RNAs, for instance 5S, 7SL RNA and U6 snRNA to be specifically co-packaged into MLV and HIV-1 particles [5,9,32]. Random RNA packaging in virions can be evaluated by monitoring the incorporation of a housekeeping mRNA abundantly expressed in cells but not enriched into virions, usually GAPDH [8] or actin mRNA [32]. The RT-qPCR can detect trace amounts of RNA and it is therefore very appropriate for obtaining such information. 

The RT-qPCR has been particularly useful to identify the packaging determinants in the viral RNA, such as the 5′ UTR structures including the Psi element, and the *trans*-acting factor, and to test the function of *trans*-acting factors participating in the assembly, such as the nucleocapsid or matrix domain of Gag protein [6,34,41,42,43,44] and Rev protein [45].

### 3.6. Advantages of RT-qPCR.

The RT-qPCR technique can be laborious to establish and needs numerous controls to guarantee accurate and reliable results. However, with careful optimization, it is highly sensitive and specific, allowing the detection of very small amounts of RNA, and the quantification of several different targets from a single RNA sample, and often from a unique (oligo-dT) RT reaction. The RT-qPCR allows to run high-throughput analysis, as numerous assays can be run simultaneously, and several targets can be quantified in the same assay (by using TaqMan probes for instance). Finally, the RT-qPCR has also the great advantage of being a safe technique, using neither radioactivity nor harmful chemicals products. In conclusion, it is the method of choice for quick and precise quantification of RNA packaging.

## 4. Fluorescence Microscopy of Fixed- or Live-Cells

Over the last few decades, fluorescence-based methods have been developed to label the RNA in cellulo with high specificity and sensitivity. While biochemical techniques based on total RNA extraction from pools of cells and viruses provide information on the average behavior of a population, fluorescence microscopy enables direct visualization of single RNA molecules within single cells and viruses. This represents a major breakthrough for the study of RNA metabolism because it accounts for cell-to-cell variation and focuses on the behavior of single RNA transcripts. Moreover, as the integrity of the sample is preserved, RNA molecules can be studied within their three-dimensional (3D) cellular environment, providing information on the spatial distribution, the surrounding molecular network and cell structure. With the live-cell fluorescence microscopy approach, a 4th dimension (time) is added and thus permits a better understanding of the dynamic nature of RNA trafficking.

Different RNA labeling techniques exist depending on whether fixed or live samples are used. In this section, we describe two techniques permitting sensitive and specific detection of single RNA molecules in cells or virions: fluorescence in situ hybridization (FISH), which consists in hybridizing multiple fluorescent DNA probes to a given RNA species in a fixed biological sample [46]; and RNA-coat protein-based labeling of live cells, which takes advantage of the specific high-affinity interaction between a fluorescently labeled bacteriophage coat protein, such as MS2, and a reporter RNA containing its cognate binding site repeated in tandem [47]. With these methods, several fluorophores are bound to the target RNA, thus providing a sufficient amount of signal for visualizing single transcripts with state of the art microscopes [46].

Recent advances in the field of fluorescence microscopy have provided a wide array of imaging tools for the study of single molecules at the subcellular scale with great spatial and temporal resolution [48]. Accordingly, the investigator will choose adequate microscopic strategies best suited to the object of study. For instance, TIRFM can be used to study viral assembly events at the plasma membrane with high signal-to-noise ratio [49,50,51], and super-resolution microscopy techniques allow accurate 3D imaging of the cell and visualization of small objects and fine structures with great resolution [15,52]. Finally, the use of advanced image analysis tools, together with mathematical and statistical analyses, allows extracting quantitative information of the acquired images with high precision. Quantitative information is a critical complement to qualitative data. It permits to measure the amount of target RNA transiting at a given subcellular localization, which can be difficult to study with biochemical approaches. For instance, quantitative FISH analyses provided important knowledge on the mechanism of MLV gRNA nuclear export by nuclear RNA export factor 1 (Tap/NXF-1) [39]. Indeed, in experiments that blocked the Tap-dependent RNA export pathway, FISH provided the high sensitivity required for gRNA detection at a very short time after transfection, when RNA splicing was still incomplete and degradation of unexported RNA molecules not yet activated [39]. Results obtained with fluorescence microscopy approaches are valuable for testing hypotheses relative to RNA transport in the cell, and they are therefore essential for developing detailed models of viral RNA packaging.

### 4.1. FISH

#### 4.1.1. Methodology

In situ RNA labeling can be used to detect endogenous or engineered RNA in fixed cells and viruses. With this technique, specific RNA transcripts are detected by hybridizing fluorescent DNA probes that are complementary to a unique RNA sequence present in the target RNA. Multiple RNA species can be distinguished using different probe sets labeled with spectrally distinct fluorophores. The specificity and stringency of hybridization needs to be optimized by adapting the temperature of hybridization, which is directly dependent on the GC nucleotide content and length of the probe, the percentage of formamide and the concentration of monovalent cations (e.g., sodium ions) that are present in the hybridization mixture. Post-hybridization washes under stringent conditions are further required to remove unbound probes, reduce background and achieve specificity [53]. FISH can be combined with immunofluorescence assays (IF) to detect proteins of interest (for instance, the retroviral Gag), and specific dyes such as 4′,6-diamidino-2-phenylindole (DAPI) or fluorescent-wheat germ agglutinin (WGA) can be also added to respectively label the nucleus and cell membranes. At the end of the procedure, coverslips containing stained samples are usually mounted in aqueous media formulated with antifading agents to preserve samples for long-term storage and prevent photobleaching.

Single-molecule sensitivity can be achieved using multiply-labeled probes [46] or many singly-labeled probes [54] that hybridize to a sequence in the target RNA, or by repeating in tandem a unique sequence of hybridization. This way, the target RNA is labeled with up to 48 fluorophores, allowing its visualization as a diffraction-limited spot that can be spatially located and counted using dedicated software [46]. Advanced algorithms have been developed in ImageJ or Matlab software to allow accurate identification of bright spots over background and localize their center position with subpixel precision in the 3D space [55,56].

#### 4.1.2. Applications of FISH in the Study of Retroviral RNA Encapsidation

**Figure 2:**
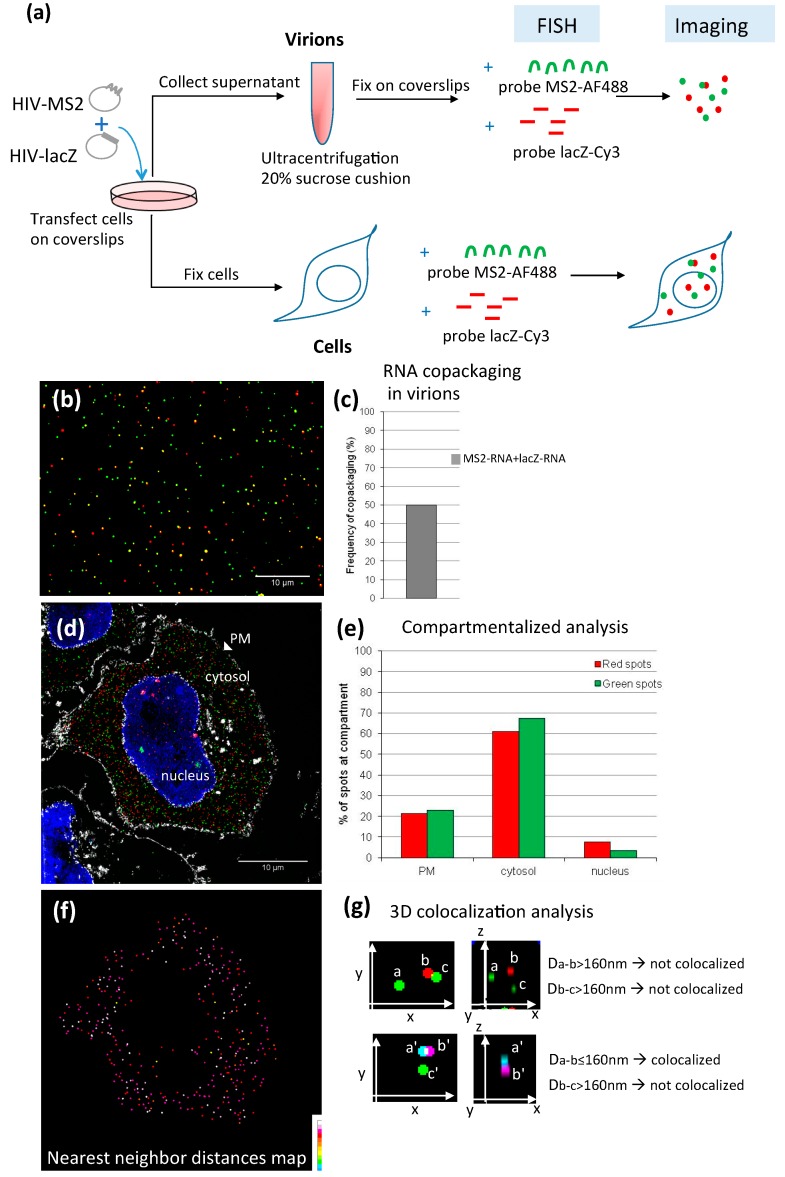
Dual-color imaging of RNA in cells and virus particles [15]. (**a**) Schematic representation of the methodology used. Cells are seeded on coverslips and transfected with plasmids encoding HIV-1 gRNA tagged either with 24xMS2 or a fragment of *lacZ* gene. Cell culture supernatants are collected and ultracentrifuged for viral particles concentration. A drop of virus-containing pellets are then allowed to settle down on coverslips and fixed for fluorescence in situ hybridization (FISH) labeling using specific probes targeting the tags. (**b**) Microscopic image of virions showing the MS2 and lacZ tagged gRNAs labeled in green and red, respectively. Colocalized spots appear in yellow. (**c**) Quantification of the percentage of virions harboring two distinctly tagged gRNAs. (**d**) 3D-structured illumination microscopy (3D-SIM) image of a cell transfected with HIV-MS2 and HIV-lacZ at 24 h post-transfection. HIV-MS2 and HIV-lacZ gRNAs are labeled in green and red by FISH, respectively. The nucleus is stained in blue using 4′,6-diamidino-2-phenylindole (DAPI) and the cellular membranes are labeled with wheat germ agglutinin, Alexa Fluor 647 conjugate (WGA-647) and shown in white. (**e**) Quantification of the distribution of red/green spots in the different compartments of the cell shown in (d) (PM for plasma membrane). (**f**) Image of the same cell as in d showing red spots colored according to the 3D Euclidian distance of each spot to its nearest neighboring spot in the green channel. This way, colocalization between red-green spots can be calculated above a threshold distance, as shown in (g). (**g**) Example of colocalization analysis between spots of different colors in the 3D space. In the upper panels, two spots may appear colocalized in 2D (*x*,*y*) but not when depth (*z*) is considered. Two spots are colocalized when the 3D-Euclidian distance (D) is ≤160 nm, as in the panels below.

The application of FISH to study the localization and trafficking of viral RNAs in cells has provided valuable insights into the mechanisms of retroviral RNA encapsidation. By introducing two different reporter sequences to tag the HIV-1 genome, like *MS2* and *lacZ* gene sequences (Figure 2), simultaneous labeling of HIV-1 RNA in two-colors is achieved using differently labeled probe sets specific for these sequences, (Figure 2), thus allowing the study of the co-packaging of two distinctly labeled RNAs into viral particles and gain insights into the mechanisms of gRNA dimerization [15]. In such experiments, virus particles need to be concentrated from cell culture supernatants, filtered through a 0.22 µm pore size and settled on gelatin-treated coverslips prior to fixation and FISH labeling. Although this method provides enough resolution to visualize single RNAs as diffraction-limited spots on a wide-field microscope, virions that contain two RNAs tagged with the same color (homodimers) are usually not resolved. Instead, two-color labeling may permit to detect the co-packaging of two differentially labeled RNAs (heterodimers). Valuable information can be obtained from two-color imaging of FISH-stained virus particles. First, by comparing the co-encapsidation frequencies of two differently tagged RNAs, it is possible to test RNA determinants and cellular locations involved in RNA packaging [15,57]. Second, one can compare the packaging efficiencies of two different RNAs. In such experiments, both RNAs must be expressed at similar levels in the cell. After recovery of virions, packaging efficiencies are calculated as the ratio of FISH spots produced by each tagged RNA [15]. Finally, by labeling Gag with a specific antibody or using a fluorescent protein fused to Gag, the proportion of virus particles containing RNA can be estimated by means of co-localization (spots of RNA colocalizing with Gag/total Gag spots). However, the amount of virus being recovered on the coverslip usually varies from one experiment to another, making it difficult to compare virus production from one cell population to another. Some promising techniques are being developed to address this issue, such as FISH coupled to flow cytometry or fluorescence fluctuation spectroscopy.

### 4.2. Live-Cell Imaging to Study Viral RNA Packaging

The principle of the MS2-system consists in the specific recognition of target sequences inserted in tandem in the RNA (i.e., MS2) by their respective RNA-binding protein, fused to a fluorescent peptide (MS2 coat protein-GFP). The MS2 system has been extensively used due to its simplicity and sensitivity. The approach has provided important insights into many aspects of the retrovirus lifecycle including transcription dynamics, nuclear export of viral RNA, translational regulation, membrane targeting, and condensation of the gRNA during virion assembly (reviewed in [58]).

The original MS2 system consists in inserting a 19-nucleotide stem-loop structure from the MS2 genome in the RNA of interest, which binds to MS2-GFP. At least three other MS2-like systems are now available, used alone or in association with the MS2-system, for live-cell RNA imaging: (i) the BglG system consists in a 29-nucleotide stem-loop recognized by the antitermination protein in the *Escherichia coli bgl* operon (Bgl system) [59]; (ii) another system is based on the coat protein of the Pseudomonas phage *PP7*, which binds its own 23-nucleotide stem-loop RNA primer-binding site (called PBS) [60]; and (iii) the third system (called λ_N_ system) has the advantage of a smaller tag insertion, and it consists in the 15-nucleotide stem loop of the antitermination signal (BoxB *RNA*) recognized by the N coat protein of bacteriophage lambda [61]. The four RNA reporter systems provide single molecule sensitivity by inserting several tag binding sites in tandem (24xMS2, 24xPP7, 18xBgl or 16xBoxB stem-loops, respectively). As for the FISH strategy, the results are critically influenced by the localization of the tag binding site in the reporter RNA. The RNA-coat protein-based labeling approach has been successfully developed for viral RNA reporters of MLV [62] and HIV-1 [57,63]. The same strategy can be adapted to image viral RNA inside virions. Cells are co-transfected with the RNA reporter system and its corresponding binding protein fused to a fluorescent protein. In order to determine the RNA packaging efficiency, a plasmid expressing fluorescent Gag proteins can be transfected to monitor the released particles (Gag) in supernatant (RNA/Gag particles). After collection of cell-culture supernatant, the virions are filtered through a 0.22 µm pore size and can be directly transferred into a glass-bottom dish, incubated 2 h with Polybrene® (hexadimethrine bromide) at 37 °C with 5% CO_2_ [57]. If required, virions can be concentrated by ultracentrifugation on 20% sucrose cushion, resuspended in Dulbecco's modified Eagle medium (DMEM) F12 before loading on gelatinized coverslips. Inverted microscope with 100× oil objective is used for epifluorescence microscopy. Image analysis is performed with algorithms (often customized) from ImageJ, Matlab or DIPImage software to reduce the background intensities in the image and achieve packaging efficiency calculation (ratio RNA/Gag) [57,63]. Interestingly, the combination of the MS2 and the BglG systems, allowing multicolor RNA imaging, has revealed that >90% of HIV-1 particles contain gRNA packaged as dimers [57].

Virion analysis can be easily associated with RNA imaging at the plasma membrane of virus producing-cells by using real-time TIRFM technologies. This latter was used to image Gag interaction with 24xMS2 gRNA during assembly of individual HIV-1 particles at the plasma membrane [63]. The pictures showed complexes formed by RNA and few Gag molecules nucleating for viral assembly. However, the approach failed to detect initial genome recognition by Gag, likely due to sensitivity limit of detection. This year, two multicolor strategies combined to TIRFM analyses have provided pictures of HIV-1 gRNA heterodimers at the plasma membrane [14,15]. Furthermore, combination of advanced 3D-structured illumination (3D-SIM) of fixed cells labeled by FISH and N&B cross-fluctuation microscopies in live cells have revealed, for the first time, that gRNA-gRNA interactions can initiate in the cytosol [15]. These promising approaches will open new lines in the investigation of the packaging of RNA species copackaged with the viral gRNA.

### 4.3. FISH versus Live-Cell Imaging

In conclusion, FISH is a powerful tool in the study of single RNA molecules within single cells and viruses. Hybridization of multiple fluorescent probes to a specific sequence in the target RNA gives high signal-to-noise ratios and bright signal providing single molecule sensitivity. The technique is therefore well suited to detect, localize and enumerate low abundant RNAs expressed in cells. A variety of fluorescent dyes exists that allow many combinations for labeling distinct populations of RNA and protein partners or cellular organelles, thus offering an appreciated level of flexibility for the imaging. Indeed, when multiple staining for RNA and/or proteins are combined (FISH/IF), valuable information on RNA–RNA/protein interactions and their spatial orchestration with the cellular structure can be obtained. Finally, organic fluorophores are more resistant to photobleaching than fluorescent proteins [64], and thus allow imaging the whole cell volume with advanced microscopy approaches such as 3D-SIM.

Both endogenous and engineered RNA can be followed using specific probes designed accordingly. The ability to label native RNA by FISH is a major advantage over live-cell imaging, as it does not require modification of the RNA in order to incorporate such tags. Since the RNA is unaltered by the binding of exogenous proteins, FISH should provide more reliable information about the localization and native state of RNA molecules. Indeed, binding of several bacteriophage proteins to the reporter RNA may affect intracellular movement and virion morphology. Nevertheless, FISH labeling requires fixation and permeabilization of cells, which can alter molecular conformations and disrupt the integrity of certain organelles or cellular structures [65]. Finally, a major advantage of live-cell imaging over FISH is that it provides time-resolved information, allowing the dynamic study of RNA trafficking and the discovery of transient interactions.

## 5. RNA Sequencing Opens New Perspectives for Studying the Virion Transcriptome

Recent breakthrough in “deep-sequencing” technologies permits exhaustive transcriptome analysis, thus facilitating the large-scale study of RNA expression and the discovery of rare transcripts. Next-generation sequencing (NGS) is based on the real-time monitoring of base extension during the DNA sequencing reaction, which is performed either on a pool of different templates (Illumina, IonTorrent) or on a single molecule (Pacbio). Depending on the platform, the data may consist either in several millions of short reads (<300 nt, Illumina, IonTorrent) or a relatively small number of very long reads (>15 kb, Pacbio), with outputs varying between 0.5 Gb and 100 Gb. Large numbers of reads permit to attain high coverage of the sample, which is instrumental for sequence assembly and for the discovery of rare molecules. During RNA sequencing (RNA-seq), the sample RNA is reverse transcribed and adapter sequences are included at the extremities of cDNA. These extra sequences are instrumental for the subsequent amplification and sequencing of the cDNA library. 

RNA-seq has been used for studying the expression of viral RNA in infected cells [66]. NGS has also been used to investigate nucleic acid content of purified virus [67,68], thus revealing different classes of transcripts present in MLV virions (including host RNA and transposable elements) [12]. The expression level of a given transcript will have a direct impact on its representation among sequencing reads, and RNA-seq thus permits to infer the relative expression of a large number of RNAs at the same time. However, very abundant molecules such as ribosomal RNA or actin mRNA may be over-represented in the reads, thus reducing the chance for detecting rare RNA in cases where the transcriptome complexity exceeds sequencing capability. High sequencing depth, in combination with RNA depletion or enrichment strategies, is therefore critical for discovering poorly expressed transcripts. Accurate quantification and comparison of RNA expression between different samples necessitate careful normalization procedure (based for instance on spike-in controls) and thorough statistical analysis [69]. At present time, top-performance RNA-seq is costly and generally beyond the reach of small laboratories, as it involves an extensive laboratory setup for library preparation, validation, and sequencing.

## 6. Conclusions

The choice of RNA quantification method in cells and virions requires consideration of several factors: sensitivity, accuracy, relative quantification, RNA size determination and probe flexibility. Each of the techniques discussed in the present review provides specific advantages and limitations to study RNA packaging. For applications in which characterization of the packaged RNAs and their relative abundance are of chief importance, the Northern blot is the more suitable choice. Indeed, the Northern blot is the only technique that provides information about RNA size. The main advantage of the RPA over the Northern blot is its higher sensitivity (10-fold), attributed by the high specific activity of the riboprobe labeling and the hybridization of the riboprobe to the target RNA in solution without prior membrane immobilization. However, recent approaches based on RT-qPCR are most commonly used. They excel for the rapid measurement of packaging efficiency and/or ability of known RNAs for which efficient sets of primers have been developed. In contrast, RNA-seq approaches are still in their infancy for the study of viral packaging but their performance opens promising perspectives. Deep sequencing of viral RNA has to deal with the representation of target molecules in the sample, and for rare transcripts it may involve the collection of significant amounts of biological material. Among all the methods discussed, RT-qPCR offers the highest sensitivity and accuracy. In practice, qPCR can detect the cDNA derived from 10 RNA copies and therefore, requires only small amounts of biological samples. Typically, either ~10% of virions released from tissue culture or 1 µg of total cellular RNA are used for the RT reaction and only a fraction (1/20 to 1/50) of the resulting cDNA is used for qPCR. Several assays can be performed with the initial sample, thus reducing the need for collecting biological material. Similar advantages are brought by the most recent imaging approaches, which permit the detection of unique tagged-RNA molecules in single cells or virions. The methods for in vivo RNA visualization provide unprecedented access to quantity, localization and dynamics of viral RNA and will likely see their usage in constant progress for the study of packaging.
